# Sodium Storage Properties of Carbonaceous Flowers

**DOI:** 10.3390/molecules28124753

**Published:** 2023-06-14

**Authors:** Xiaolei Sun, Feng Luo

**Affiliations:** School of Materials Science and Engineering, Smart Sensing Interdisciplinary Science Center, Tianjin Key Lab for Rare Earth Materials and Applications, Center for Rare Earth and Inorganic Functional Materials, Nankai University, Tianjin 300350, China

**Keywords:** lithium-ion batteries, sodium-ion batteries, N/O heteroatoms, hierarchical porous structures, anode materials, ultralong cycling lifetimes, high-rate capabilities

## Abstract

As a promising energy storage system, sodium-ion batteries face challenges related to the stability and high-rate capability of their electrode materials, especially carbon, which is the most studied anode. Previous studies have demonstrated that three-dimensional architectures composed of porous carbon materials with high electrical conductivity have the potential to enhance the storage performance of sodium-ion batteries. Here, high-level N/O heteroatoms-doped carbonaceous flowers with hierarchical pore architecture are synthesized through the direct pyrolysis of homemade bipyridine-coordinated polymers. The carbonaceous flowers could provide effective transport pathways for electrons/ions, thus allowing for extraordinary storage properties in sodium-ion batteries. As a consequence, sodium-ion battery anodes made of carbonaceous flowers exhibit outstanding electrochemical features, such as high reversible capacity (329 mAh g^−1^ at 30 mA g^−1^), superior rate capability (94 mAh g^−1^ at 5000 mA g^−1^), and ultralong cycle lifetimes (capacity retention rate of 89.4% after 1300 cycles at 200 mA g^−1^). To better investigate the sodium insertion/extraction-related electrochemical processes, the cycled anodes are experimentally analyzed with scanning electron microscopy and transmission electron microscopy. The feasibility of the carbonaceous flowers as anode materials was further investigated using a commercial Na_3_V_2_(PO_4_)_3_ cathode for sodium-ion full batteries. All these findings indicate that carbonaceous flowers may possess great potential as advanced materials for next-generation energy storage applications.

## 1. Introduction

The widespread use of lithium-ion batteries (LIBs) in a diverse range of applications, including portable electronics and electric vehicles, has led to growing concerns regarding the availability of lithium resources [1,2,3]. This is particularly worrisome given the escalating demand for lithium, coupled with its limited reserves and uneven distribution across the planet. The utilization of alternative battery systems has become a topic of interest due to their potential to offer high energy density and abundance in natural reserves [3,4,5]. This has led to an increased focus on exploring their potential in various fields including power grid systems. During the past decade, numerous studies have highlighted the potential of sodium-ion batteries (SIBs) as a viable substitute for LIBs owing to the abundant availability of sodium sources [6,7,8]. The cost-effective production of SIBs, compared with LIBs, can be attributed to the ample reserves of sodium resources. The present research focuses on the working principle of SIBs, which is akin to a rocking-chair mechanism and is based on the insertion and extraction of alkali cations into battery electrode materials [5]. This mechanism allows for the transfer of material design knowledge from LIBs to SIBs. In comparison to LIB systems, SIBs possess a low reduction potential of −2.71 V vs. standard hydrogen electrode (SHE), which is in close proximity to that of lithium (−3.02 V vs. SHE) [9]. This characteristic of SIBs ensures a wide voltage window and high energy density. The utilization of Na^+^ ion charge carriers presents a promising avenue for reducing the costs of SIB systems through the development of novel chemistry and materials. The inability of sodium to form an alloy with aluminum has led to the adoption of aluminum foil as a current collector for anodes, thereby circumventing the issue of over-discharge [4,10]. Therefore, SIBs have the potential to supplant lithium-ion technology as the preferred battery chemistry that energizes our civilization in the foreseeable future [7,9].

The use of SIBs presents a promising prospect; however, the progress of SIBs is impeded by a significant obstacle in the identification of appropriate electrode materials that can endure the stable accommodation of Na^+^ ions over extended cycles. In contrast to the LIB system, Na^+^ has a larger ionic radius (1.02 Å) than Li^+^ (0.76 Å) [11,12]. This disparity may result in hindered ion diffusion, significant volumetric expansion, and more pronounced structural distortion of the electrode materials during the reversible sodiation/desodiation processes. Graphite is a commonly used anode material for LIBs. However, its performance in sodium-ion storage is limited owing to the formation of graphite intercalation compounds NaC_64_ instead of thermodynamically unstable NaC_6_ when using ester electrolytes [13]. This results in a low reversible capacity of less than 20 mAh g^−1^. Numerous anode materials, such as metal oxides, nitrides, metals, and nonmetals, have been well-documented as potential options for enhancing the anodic performance of SIBs [14,15,16,17]. Nonetheless, owing to their elevated redox potentials, inadequate cycling stability, deficient initial Coulombic efficiency, and substantial polarization during Na^+^ insertion/extraction reactions, they have been deemed unsuitable for prolonged operations.

Therefore, it is essential to develop SIB anodes that are more appropriate and work better electrochemically. Due to their low working potential and adequate capacity, carbonaceous materials have been investigated as SIB anodes. To date, numerous carbon products with diverse microstructures have been developed, including carbon nanotubes, nanofibers, hollow nanospheres, graphene, porous carbon, and their hybrid composites [18,19,20,21]. Currently, amorphous carbon compounds may be the most effective option among all the anode possibilities for SIBs, taking into account their electrochemical properties [22]. Due to the irregular structure of hard carbon, also known as nongraphitic carbon, which facilitates Na^+^ ion insertion and extraction, it has been widely studied for sodium storage [7]. Previous investigations have shown the encouraging performance of hard carbon as an anode for SIBs, with low average potentials and high specific capacities [23,24,25]. Although significant progress has been achieved, further work should still be carried out to enhance the cycle and rate capacity.

An alternative method for enhancing the electrochemical cyclic performance of carbon-based anodes involves the introduction of non-carbon elements, such as oxygen (O), nitrogen (N), boron (B), fluorine (F), and phosphorus (P), to modify the surface functional groups [26,27,28,29,30]. The incorporation of heteroatoms into the carbon frame has been found to augment both the electrochemical reactivity and electrical conductivity, resulting in an enhanced capability for sodium storage. For example, N-doping can produce external flaws in carbon matrices, which can improve the storage capacity and electrical conductivity of the materials [31]. N-doped carbon products with different morphologies, including nanowires, nanosheets, and microspheres, have recently demonstrated significantly improved electrochemical battery performance [32,33,34,35]. Creating a permeable framework to explore some open pathways for Na^+^ ions flow and storage is another successful approach [36]. To be more precise, strengthened porous carbon frame construction with increased surface area and pore volume could provide large diffusion pathways for fast mass transport and buffer the enormous volume strain, bringing advantages in improving the electrochemical performance of SIBs [21,36]. Thus, in the context of carbon-based anode materials, the presence of a porous nanostructure and the integration of heteroatoms are considered advantageous for storing more Na^+^ ions. Despite the fact that these efforts have made considerable progress, carbonaceous electrodes can still be improved in terms of rate capacity and cyclic stability [14,15,37]. Finding an effective and instructive strategy to prepare hierarchically porous carbon loaded with heteroatoms might therefore improve the energy efficiency of sodium storage [38].

In this work, we present a facile approach for fabricating high-level N/O heteroatoms-doped carbonaceous flowers with hierarchical pore architecture through the direct pyrolysis of homemade bipyridine-coordinated polymers. The as-prepared carbon flowers could provide effective transport pathways for electrons/ions, thus allowing for remarkable sodium-ion storage merits. When employed as the anode materials for SIBs, the resulting carbon products exhibit outstanding electrochemical features, such as high reversible capacity (329 mAh g^−1^ at 30 mA g^−1^), superior rate capability (94 mAh g^−1^ at 5000 mA g^−1^), and ultralong cycle lifetimes (capacity retention rate of 89.4% after 1300 cycles at 200 mA g^−1^), which could be compared with those of other carbon-based anodes reported previously. In addition, in-depth post-morphology characterizations of the cycled electrodes are experimentally carried out to investigate the sodium insertion/extraction-related electrochemical processes. Furthermore, a carbon flowers/Na_3_V_2_(PO_4_)_3_ full cell also exhibits high capacity and stable cycling performance. All these findings indicate that carbonaceous flowers may possess great potential as advanced electrodes for next-generation energy storage applications.

## 2. Results and Discussion

**Figure 1:**
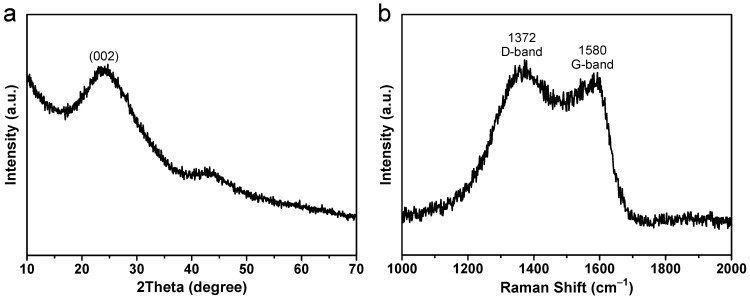
(**a**) XRD pattern and (**b**) Raman spectrum of the as-obtained carbon samples.

The generation of carbon products with abundant defects was achieved through the controlled carbonization of a novel set of bipyridine-coordinated polymers at 650 °C, as outlined in the experimental section. The X-ray powder diffraction (XRD) spectrum of the as-prepared carbon samples is illustrated in Figure 1a. The observed pattern exhibits two distinct and prominent broad peaks located at 2θ angles of 24° and 43°. The degree of expansion is attributed to the amorphous structural characteristics. It exhibits two distinct peaks, with the first peak at around 24° being attributed to the (002) plane, which corresponds to the amorphous carbon peak. The second peak at about 43° is mainly attributed to the graphitic nature of the carbon, resulting in the indistinguishability of the (100) and (101) planes. Evidently, the broadness of the peak at approximately 24° is conspicuous in comparison to that at about 43°, probably due to the distortion induced by defects of N and O heteroatoms.

Raman spectroscopy is widely regarded as an optimal analytical technique for the examination of carbon-based materials owing to its exceptional sensitivity in distinguishing between graphitic and amorphous carbon structures. Figure 1b depicts the Raman spectrum of the specimens under ambient conditions. In general, the Raman spectrum of carbonaceous materials exhibits two prominent peaks, which are commonly referred to as the D and G bands. The D-band, which occurs at approximately 1372 cm^−1^, is attributed to the A_1g_ vibration mode of the sp^3^-hybridized carbon aromatic rings that arises from the existence of defects or disorders caused by O/N doping [39]. Conversely, the G-band, which occurs at approximately 1580 cm^−1^, corresponds to the E_2g_ mode of the C-C bond stretching of sp^2^ hybridized C-atoms and is indicative of the presence of ordered graphitized sp^2^ carbon in specimens [40]. Moreover, the Raman spectroscopy technique exhibits a high degree of sensitivity to even the slightest structural variations present in carbon-based materials. Typically, the *I*_D_/*I*_G_ ratio of integrated intensity (area) could serve as a valuable indicator for evaluating the level of disorder in carbon-based materials [41]. A high ratio of *I*_D_ to *I*_G_ suggests the production of significant quantities of defects. Regarding the obtained carbon samples, the calculated *I*_D_/*I*_G_ value is 2.5, which is higher than that of the carbon nanosheets (~1.2) [42] and graphene oxide (~2.2) [43], signifying a reduced level of graphitization in the carbon microstructures, characterized by abundant disordered structures and imperfections. In addition, the increased applicability of carbon materials as anodes for sodium storage is ascribed to the presence of defects or disorders, which has been shown to enhance the slope capacity in the following discharge/charge profiles. This is due to the binding of Na^+^ ions to the structural defects of the carbonaceous materials.

**Figure 2:**
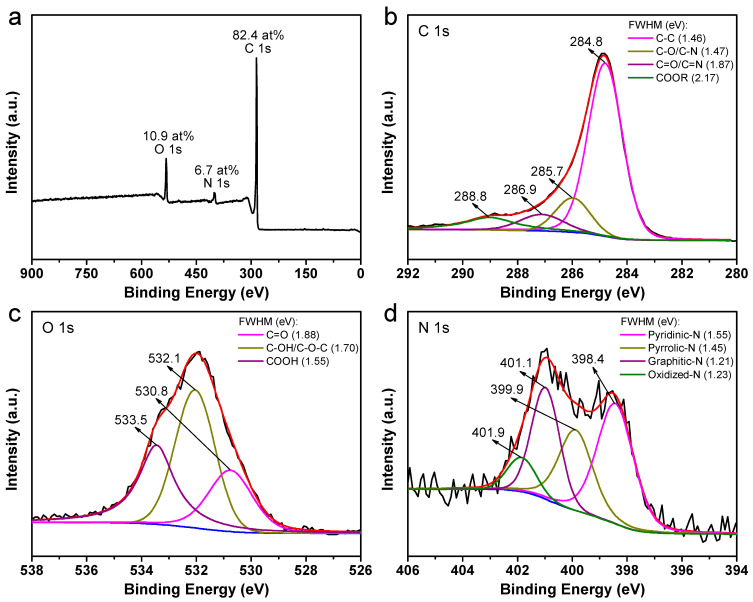
(**a**) XPS survey spectrum, and high-resolution XPS spectra of (**b**) C 1s, (**c**) O 1s, and (**d**) N 1s of the as-prepared carbon samples. The black line is the original data; the red line is the fitted data; and the blue line is the background.

Subsequent X-ray photoelectron spectroscopy (XPS) analysis was conducted to elucidate the chemical composition of the as-obtained carbon materials. Figure 2a displays a wide-range XPS survey spectrum that provides evidence of the presence of carbon (C), oxygen (O), and nitrogen (N) without any indication of impurities. The peaks observed at approximately 284.6 eV, 532 eV, and 400.4 eV correspond to these elements. The chemical composition of the carbon product reveals that it comprises 82.4 at% carbon, 10.9 at% oxygen, and 6.7 at% nitrogen. It can be deduced that there is a lower rate of nitrogen atom transfer because the N:C ratio is 0.1 compared to 4,4′-bipyridine molecules (N:C = 0.2). Generally speaking, the hydrophilicity of carbonaceous materials can be characterized by the O/C ratio; the higher the ratio is, the stronger the hydrophilicity is. Moreover, (N + O)/C represents the number of polar functional groups; the higher the ratio is, the more polar the carbon material is. Based on the above component analysis, the calculated values of the surface hydrophilicity (~0.13) and polarity (~0.21) are both high, which could facilitate the fast adsorption and immersion of electrolyte on the surface of the electroactive carbon, thus improving electrode reaction kinetics. In addition, the peak binding energy positions and full-width-half-maximum (FWHM) of the corresponding species agree with the values found in the literature [44,45]. In Figure 2b, it is observed that the high-resolution C 1s core level spectrum can be deconvoluted into four distinct peaks. Specifically, these four peaks correspond to the following chemical species: C-C (~284.8 eV), C-O/C-N (~285.7 eV), C=O/C=N (~286.9 eV), and -COOR (~288.8 eV) [46]. Based on the high-resolution O 1s core-level spectrum depicted in Figure 2c, it can be inferred that three distinct oxygen-containing functionalities are present, namely, C=O (~530.8 eV), C-OH/C-O-C (~532.1 eV), and COOH (~533.5 eV) [47]. The N 1s spectrum shown in Figure 2d can be assigned to four distinct nitrogen species, namely pyridinic-N (~398.4 eV), pyrrolic-N (~399.9 eV), graphitic-N (~401.1 eV), and oxidized-N (~401.9 eV) groups [48]. It is generally accepted that the enhancement of pseudocapacitance in battery electrodes through oxidation/reduction is a well-established phenomenon, particularly with respect to the presence of oxygen-containing functionalities, specifically C-C on carbon [21]. Additionally, the presence of pyridinic-N and pyrrolic-N active species confers advantageous wettability properties. The pyrrolic-N and graphitic-N functional groups have the potential to serve as primary active sites, thereby facilitating electron transfer and augmenting the sodium storage capacity [46]. The present study reveals that the carbon samples exhibit a significant presence of C=O (23.7 at%), pyridinic-N (39.9 at%), pyrrolic-N (25.6 at%), and graphitic-N (26.4 at%). These fascinating structural characteristics are conducive to facilitating fast charge-transfer reactions and serving as abnormal sodium-ion storage sites, ultimately leading to enhanced electrochemical behaviors [3].

**Figure 3:**
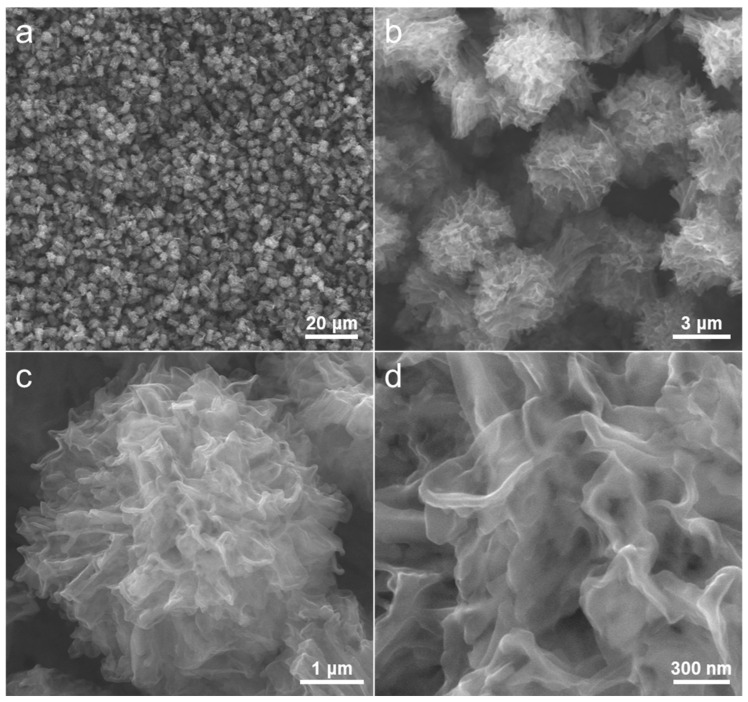
(**a**–**d**) Representative SEM images of the as-synthesized hierarchical flower-like carbon microstructures under different magnifications.

The morphological characteristics of the carbon samples were initially assessed through scanning electron microscope (SEM) analysis. The resulting morphologies of the carbon materials are depicted in Figure 3, showcasing their typical SEM images at different magnifications. The low magnification SEM images, as illustrated in Figure 3a,b, reveal that the carbon structures, which are both uniform and hierarchical in nature, exhibit random aggregation and overlap with one another. Furthermore, it is evident from the SEM images at high magnification (Figure 3c,d) that the carbon products exhibit flower-like structures with a diameter of approximately 4 μm. The architecture comprises numerous carbon lamellae possessing uneven surfaces, which are interconnected and interlinked to create a distinctive bath flower-shaped structure. In recent years, hierarchical microstructures have garnered significant attention and interest within the field of architecture due to their distinct advantages and supplementary mechanisms [49,50,51]. These advantages include small-size effects, interfacial effects, and quantum confinement effects [52]. In addition, the flake structure that is linked together has the potential to facilitate efficient charge transfer and minimize transmission resistance within carbon materials [53].

**Figure 4:**
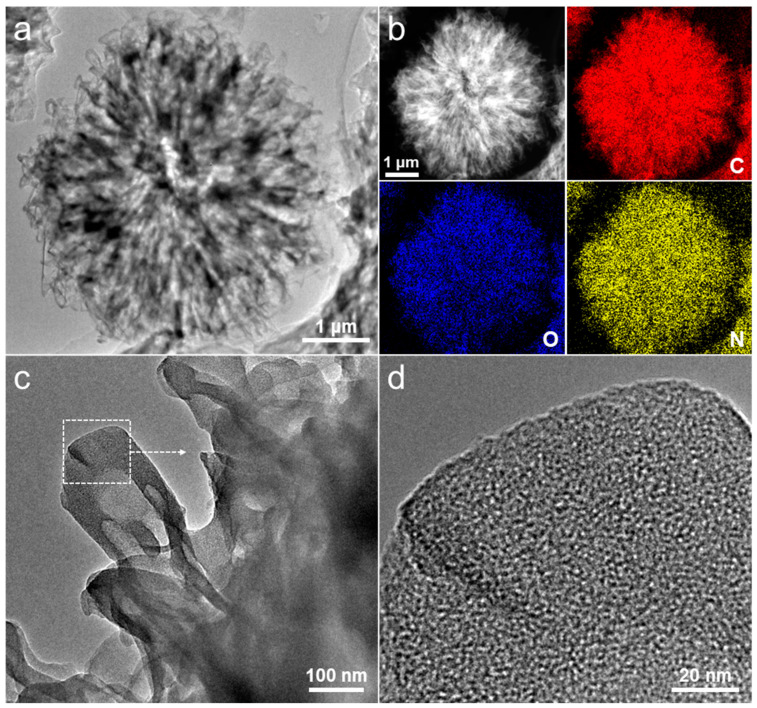
(**a**) TEM and (**b**) dark-field scanning TEM images of a single carbon microflower, and the corresponding elemental mapping images of carbon, oxygen, and nitrogen. (**c**) TEM and (**d**) high-resolution TEM images of an individual nanoflake (zoom in of marked square region in (**c**).

Transmission electron microscope (TEM) observations were utilized to systematically investigate the microstructure and composition of the carbon products that were prepared. The TEM and dark-field scanning TEM images of the synthesized carbon flower on a Cu grid (Figure 4a–c) reveal that the micrometer-sized carbon specimen retains its unique bath flower-like configuration, which is composed of aggregated nanoflakes. The uniform distribution of carbon, oxygen, and nitrogen elements in the flower-like carbon sphere is demonstrated in the accompanying elemental mapping images, as depicted in Figure 4b. According to the energy dispersive X-ray (EDX) analysis, the atomic composition of carbon, oxygen, and nitrogen in the as-synthesized carbon materials is 84.1 at%, 9.7 at%, and 6.2 at%, respectively. The outcome closely aligns with the surface compositions provided in the XPS survey spectrum (Figure 2a), thereby implying the homogeneity of the atom distribution across the whole area of the carbon flower. The high-resolution TEM image in Figure 4d further reveals that large quantities of micropores are homogeneously distributed within the carbon nanoflakes. The uniform micropores should originate from the combustion of bipyridine-coordinated polymers during the pyrolysis process. This result can also be confirmed by the following nitrogen adsorption analysis. On the whole, such microflowers, via three-dimensional assembly through intersecting nanoflake support of, have particular structural advantages as electrode materials, including easily accessible active sites, nanoscale diffusion channels of electrolytes, facile strain relaxation with large surface-to-volume ratios, and consecutive conducting pathways [14].

**Figure 5:**
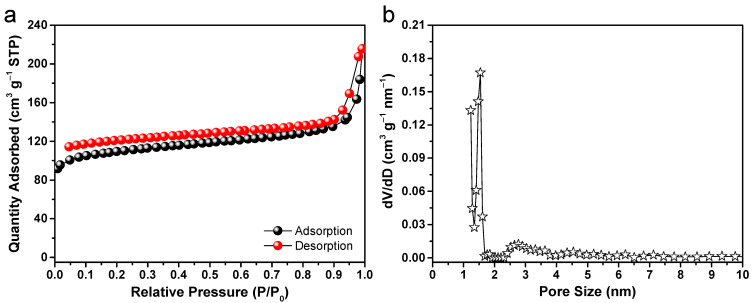
(**a**) Nitrogen physisorption isotherm, and (**b**) the corresponding pore size distribution curve obtained from the adsorption branch for the hierarchically porous carbon microflowers.

Nitrogen adsorption/desorption measurements of the hierarchical carbon flowers were performed in order to check their porous characteristics, such as Brunauer–Emmett–Teller (BET) specific surface area and pore size distribution. The isotherm for adsorption/desorption is manifested in Figure 5a, accompanied by its corresponding pore size distribution that is obtained from the adsorption segment of the isotherm curve. The analysis reveals that the samples display a type II isotherm accompanied by a hysteresis loop, which suggests the presence of a significant amount of porous structure. The sudden increase in adsorption uptake observed at high relative pressures (P/P_0_ > 0.9) is attributed to the existence of macropores, which is consistent with the findings from SEM and TEM analyses. Figure 5b plots the results of a pore size analysis obtained from the adsorption branch by applying the nonlocal density functional theory (NLDFT) method [54]. It is evident that the carbon microflowers possess small mesopores (2~6 nm) and a significant concentration of micropores (1~2 nm). The observed porous configuration results in a notable BET specific surface area of approximately 357 m^2^ g^−1^ for the prepared carbon flowers. Usually, anode materials for sodium-ion storage can benefit from both their porous nature and high specific surface area. These beneficial characteristics can increase the exposure of active sites, improve mass transfer and electrolyte adsorption, and mitigate volume changes, thus leading to significant enhancement of the electrochemical performance.

**Figure 6:**
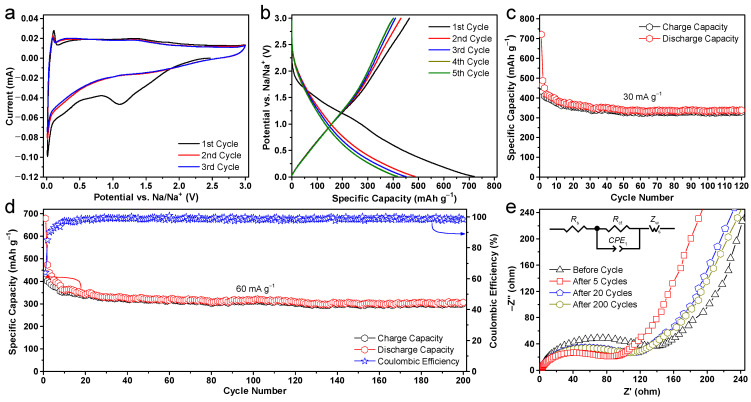
Electrochemical performance of the hierarchically porous carbon flowers electrodes. (**a**) The initial three-cycle CV profiles at a scan rate of 0.1 mV s^−1^ within the potential range of 0.01–3.0 V vs. Na/Na^+^. (**b**) Galvanostatic discharge/charge voltage profiles plotted for the initial three cycles, and (**c**) cycling performance at a small current density of 30 mA g^−1^ in the potential window of 0.01–3.0 V. (**d**) Discharge/charge cycling behaviors and the corresponding Coulombic efficiency plots at a high current density of 60 mA g^−1^ with a voltage range of 0.01–3.0 V. (**e**) Electrochemical impedance Nyquist plots before cycling and after 5, 20, and 200 cycles in (**d**) with a frequency sweep from 10^5^ to 10^−2^ Hz. Inset shows the equivalent circuit model of the studied systems.

The sodium-ion storage behaviors of the as-prepared hierarchical carbon flowers were studied through cyclic voltammetry (CV) and galvanostatic discharge/charge cycling measurements. The CV curves of the carbon specimens within the voltage range of 0.01–3.0 V vs. Na/Na^+^ are depicted in Figure 6a, by which Na^+^ insertion/extraction reactions were investigated. During the initial discharge process, a reduction peak at around 0.9 V is detected, which subsequently vanishes in the following scans. This phenomenon is believed to be attributed to the decomposition of the electrolyte and the consequent formation of an inevitable solid electrolyte interphase (SEI) layer [6]. Moreover, two redox peaks are discernible in the lower potential range (approximately 0 V), resembling the process of insertion of sodium ions into carbon materials. These peaks usually correspond to the insertion and extraction of sodium ions in the interlayer of the graphitic microcrystallites. The weak redox peaks and the rectangular shape of the CV curves over a broad voltage range at high potential suggest that the storage of sodium ions displays certain characteristics that resemble those of capacitive behavior [37]. It can be deduced that sodium-ion adsorption and desorption could occur on the surface of carbon flowers through physical sorption and redox reactions that involve N/O-containing functional groups and/or defect sites [46]. The CV curves demonstrate a high degree of overlap, suggesting that the electrodes composed of carbon flowers exhibit favorable stability and reversibility in relation to sodium storage. The observed variation in the initial cycles could potentially be attributed to the formation of the SEI layer and localized structural reorganization within the electrode, which can serve to mitigate the deformation and stress incurred during sodiation/desodiation processes. The electrochemical stability of the carbon flowers electrode was further investigated through galvanostatic cycling testing. The discharge/charge profiles for the carbon flowers obtained at a current density of 30 mA g^−1^ within the potential range of 0.01 to 3.0 V vs. Na/Na^+^ are depicted in Figure 6b. In the initial cycle, the electrode composed of carbon flowers exhibits discharge and charge capacities of 721 and 465 mAh g^−1^, respectively, resulting in an initial Coulombic efficiency of 64.5%. Generally, the occurrence of a low initial Coulombic efficiency is a prevalent phenomenon in high-surface-area carbon materials. This is primarily attributed to the formation of the SEI layer, surface reactions on the electrode materials, and the permanent entrapment of sodium ions at active sites and/or graphitic interlayers. These observations align with the broad peaks detected during CV scans. Enhancement of the low initial Coulombic efficiency can be achieved through the regulation of the specific surface area, refinement of the binder, and alteration of the surface of carbon materials [55]. Upon completion of the initial cycles, the carbon flower electrode attains a stable charge capacity of around 401 mAh g^−1^. The prolonged cycling performance of the carbon flowers electrode is further illustrated in Figure 6c, wherein a current density of 30 mA g^−1^ was applied. The initial cycles exhibit a notable decrease in capacity, potentially attributable to the incomplete and undeveloped SEI layer on the carbon materials, as well as the potential entrapment of sodium ions in some defective sites. It is noteworthy that the cycling stability has the potential to be improved by optimizing the electrolyte used in the current research. Furthermore, it can be seen that a high reversible specific capacity of 329 mAh g^−1^ is achieved after 120 cycles, relating to a moderate capacity retention of 76.3% compared to the specific capacity of the second cycle. In comparison with previous reports on carbon nanostructures, the present carbon flower anode shows a higher capacity value (~340 mAh g^−1^) on average. As displayed in Figure 6d, the carbon flowers electrode also exhibits excellent long cycling performance and reversibility when the current density increases to 60 mA g^−1^. Specifically, the Coulombic efficiency of the second cycle surpasses 84.9% and experiences a swift rise to above 97.5% after 10 cycles, which suggests favorable reaction reversibility and structural stability. Following 200 cycles of deep discharge and charge, the electrode composed of carbon flowers demonstrates a high reversible capacity of 301 mAh g^−1^. Apparently, it is observed that the capacity of the carbon flowers electrode increases from 80 to 110 cycles. The activation step may originate from the delayed infiltration of the electrolyte into the micropores.

Moreover, to determine the electrode kinetics during cycling, the cell impedances were measured prior to cycling and subsequent to a specific number of cycles. As illustrated in Figure 6e, the impedance Nyquist plots demonstrate a notable enhancement in capacitive-like characteristics, as indicated by the more pronounced upward slope of the Z′–Z″ curves at lower frequencies. The proposed equivalent circuit model (inset) consists of surface film (*R*_s_) and charge transfer (*R*_ct_) resistances, a constant phase element (*CPE*_1_), and Warburg diffusion impedance (*Z*_w_). Obviously, the charge transfer resistance of the new electrode exhibits the highest value, owing to the absence of any prior discharge/charge cycles. Following the initial cycle, the charge transfer resistances of the electrode experience a significant decrease (~88.7 Ω), resulting in notably lower values compared to that of the pristine cell (~143.1 Ω). After that, the whole cell resistance gradually increases in the subsequent cycles, which could be attributed to the continuous formation of the SEI layer during cycling [56]. While one can see that the cell impedance spectra measured after 20 and 100 cycles exhibit similar semicircle radii and *R*_ct_ values (~113.4 and ~112.4 Ω) that indicate no significant change in the impedance of the carbon flowers electrode. The observed feature could potentially be linked to the activation of surface and lattice expansion mechanisms, which facilitate the transportation of sodium ions. These findings indicate that the carbon flowers electrode exhibits negligible resistance alteration upon repeated cycling, thereby validating the durability of the SEI layer formed during the initial dozens of cycles. In principle, the observed phenomenon would lead to an increase in the rate of electron kinetics within the carbonaceous electrode, consistent with the results obtained from the CV and galvanostatic discharge/charge tests shown in Figure 6.

**Figure 7:**
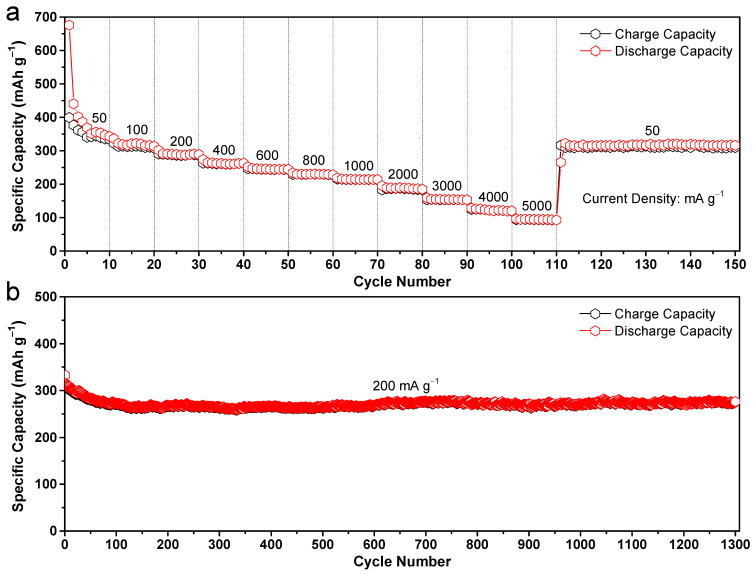
(**a**) Rate performance of the hierarchically porous carbon flowers electrodes with varying current densities from 50 to 5000 mA g^−1^, and (**b**) a long cycle life test at a high current density of 200 mA g^−1^ after three CV activation cycles at 0.1 mV s^−1^ between 0.01 and 3.0 V.

The parameter of rate capability holds significant importance for a multitude of applications that necessitate expeditious charging. To ascertain the aforementioned attribute of the hierarchically porous carbon flowers, galvanostatic discharge/charge measurements were further conducted at different enhanced current densities spanning from 50 to 5000 mA g^−1^ for 150 cycles. The electrode was cycled ten times at each high current density. As illustrated in Figure 7a, the electrode made of carbon flowers exhibits reversible capacities of 341, 313, 286, 261, 244, 230, 214, 188, 153, and 122 mAh g^−1^ at current densities of 50, 100, 200, 400, 600, 800, 1000, 2000, 3000, and 4000 mA g^−1^, respectively. The anode exhibits Coulombic efficiencies exceeding 98% throughout multiple cycles, even when subjected to both low and high current densities. It is a widely observed and inevitable phenomenon in SIBs that their specific capacities tend to decrease as the current densities increase. The utilization of high current densities restricts the electron transport phenomena within the electrode to a short discharging/charging duration, thereby inducing electrode polarization and consequently causing a decline in sodium storage capacity. Remarkably, the virtual reversible capacity of 94 mAh g^−1^ remains intact despite a 100-fold increase (i.e., 5000 mA g^−1^) and an increase in the current density of discharge/charge. To the best of our knowledge, the sodium storage performance significantly outperforms other carbonaceous anodes [14,20,22,23,33]. This characteristic is highly sought after for high-power battery applications. Following a reduction in current density to its initial value of 50 mA g^−1^, the specific charge capacity is observed to recover to 310 mAh g^−1^ with a high capacity retention as high as 100% even after an additional 40 cycles. It is noteworthy that the observed value closely approximates the initial level of the first 10 cycles under identical current density. Following a significant discharge current density, the reversible capacity exhibits a prompt restoration to its initial value, thereby demonstrating the exceptional rate capability of the carbon anode. The above observations unambiguously indicate that the acquired carbonaceous flowers exhibit remarkable stability, even when subjected to conditions of ultrahigh current densities.

Figure 7b illustrates the long-term discharge/charge cycling stability of the electrode composed of hierarchically porous carbon flowers, which was conducted at a high current density of 200 mA g^−1^. It is important to note that the cell was subjected to pre-activation via a CV measurement procedure, wherein a low scan rate of 0.1 mV s^−1^ was employed for three cycles to guarantee complete activation of the anode electrode. During the initial 100 cycles, the battery capacity experiences a slight decrease from 310 to 270 mAh g^−1^ by the 100th cycle. However, in the subsequent cycles, the capacity gradually increases and maintains a high level of capacity. Finally, the electrode exhibits a significant reversible capacity of 277 mAh g^−1^ even after 1300 deep cycles, resulting in a notable capacity retention rate of 89.4% and a low capacity-degradation rate of 0.008% per cycle. The increase in capacity observed after 100 cycles could be attributed to the activation process, as evidenced by the decrease in charge transfer resistances in Figure 6e. This phenomenon is commonly observed in various structured carbon electrodes [14,15]. The results indicate that the hierarchically porous carbon flowers electrode possesses exceptional reversibility characteristics, thereby corroborating its ability to undergo significant expansion and contraction while maintaining its structural integrity.

**Figure 8:**
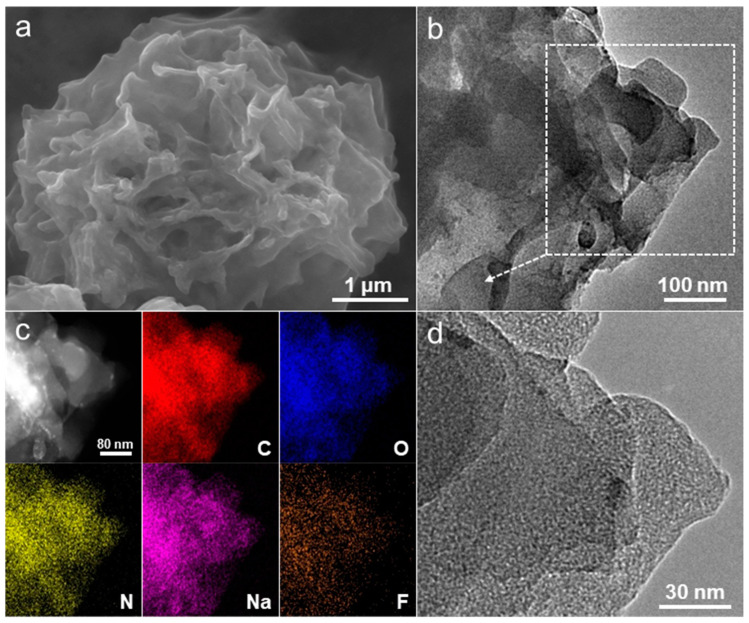
(**a**) SEM, (**b**) TEM, (**c**) dark-field scanning TEM with the corresponding elemental mapping, and (**d**) high-resolution TEM images of a single carbon microflower after 200 deep discharge/charge cycles at a current density of 60 mA g^−1^.

To comprehensively investigate the structural characteristics of the cycled carbon microflower anodes after repeated discharge/charge processes, the cells that were subjected to 200 cycles at a current density of 60 mA g^−1^ were initially accessed within an argon-filled glovebox. Subsequently, the extracted electrodes underwent multiple cleaning cycles using DMC solvent, followed by an overnight drying period to eliminate any remaining NaPF_6_ components. The analysis of the post-morphological characteristics was conducted using SEM and TEM techniques. The results depicted in Figure 8a,b indicate that the carbon microflower exhibits minimal alterations following 200 deep cycles, thereby indicating the robust structural durability of the electrode throughout the cycling process. In comparison to the findings presented in Figure 3c, it is observed that certain gelatinous coatings on the carbon surface appear to be less distinct. This phenomenon may be attributed to the existence of the roughened SEI layer on the electrode surface. This discovery aligns with the expected outcome following a prolonged period of experimentation that encompassed numerous iterations. The findings exhibited in Figure 8c demonstrate the utilization of dark-field scanning TEM to capture the image of a single nanoflake, alongside elemental mapping images that establish a correlation between the distribution of sodium and fluorine within the carbon nanoflake during cycling. These results provide evidence that the as-formed SEI layer fully encompasses the surface of the carbon flower electrode that underwent cycling. Furthermore, the high-resolution TEM image depicted in Figure 8d illustrates that the hierarchically porous structure remains largely unaltered even after undergoing 200 deep discharge/charge cycles. This observation suggests that the structure maintains its original properties despite long cycling. The carbon flower anode is hypothesized to possess the capacity to substantially mitigate the occurrence of significant cracking during cycling, owing to the comparability of its shapes before and after cycling. This guarantees that their electrochemical performance is outstanding. In addition, it is imperative to consider surface functional groups when assessing the electrochemical properties of a substance, as they have a significant impact on the wetting behaviors of both the electrolyte and the material surface.

**Figure 9:**
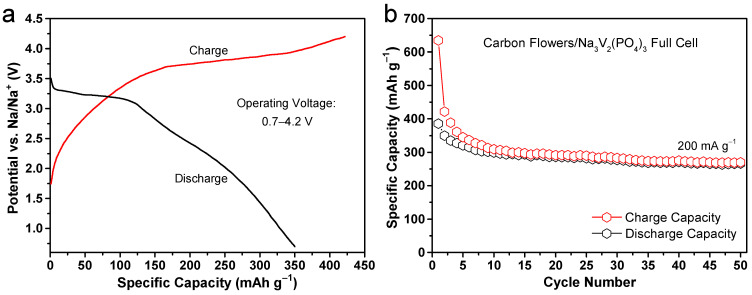
(**a**) Typical charge/discharge profiles, and (**b**) cycling performance of the carbon flowers/Na_3_V_2_(PO_4_)_3_ full cell at a current density of 200 mA g^−1^ between 0.7 and 4.2 V.

Following the inadequacy of sodium metal as a proficient sodium ion reservoir, it is imperative to conduct an assessment of carbonaceous flowers in a full battery cell. In order to accomplish this objective, rechargeable SIBs were constructed through the interconnection of the carbonaceous flowers anode with a commercially available Na_3_V_2_(PO_4_)_3_ cathode. The second charge/discharge curves of the full battery are illustrated in Figure 9a, which were measured at a constant current density of 200 mA g^−1^ based on the mass of the carbon flowers anode over the voltage range from 0.7 to 4.2 V. The electrochemical processes taking place within the test battery are indicated by the charge and discharge plateaus, which correspond to the reversible sodiation and desodiation behaviors [57]. The full cell exhibits charge and discharge specific capacities of 422 and 350 mAh g^−1^, respectively, resulting in a Coulombic efficiency of up to 82.9%. Figure 9b further displays the cycling performance of the full cell. As expected, the carbonaceous electrode manages to maintain a significant reversible capacity of about 266 mAh g^−1^ after 50 cycles, indicating remarkable cycling stability. These results therefore confirm the highly reversible sodium storage behaviors of the carbonaceous flowers as advanced anode materials in energy applications.

To sum up, the exceptional electrochemical performance of the as-prepared carbon flowers for sodium-ion storage can be attributed to their distinctive architecture, which is characterized by a large surface area of approximately 357 m^2^ g^−1^, high oxygen and nitrogen contents up to 17.6 at%, and numerous micropores ranging from 1 to 2 nm. The aforementioned effective and instructive approach motivates the forthcoming nanostructure development of operative carbon substances. Additionally, theoretical simulations and advanced in situ characterization techniques should be employed to investigate the sodium-ion storage mechanism and to better understand how carbonaceous flowers are related to electrochemical characteristics [57,58]. This will help improve the design of carbonaceous electrode materials and make them more efficient. In particular, the establishment of a chemically and mechanically stable passivating SEI layer during the first sodiation process is of paramount importance for ensuring the continued stability of the anode [59]. Hopefully, the results of these experiments will serve as inspiration and pathways for the development of future high-performance sodium-ion storage materials with senior microstructures.

## 3. Materials and Methods

### 3.1. Materials Preparation

Without any additional purification, all compounds were used directly as they were given. Typically, a 2 L vessel was used to make carbon precursors. Typically, 100 mL of 0.1 M 4,4′-bipyridine water-ethanol solution (volume ratio: 1:15) was used to dissolve 1.0 g of triblock copolymer (pluronic F127). This solution is referred to as solution A. Another solution, designated solution B, contained 0.056 M CuCl_2_·2H_2_O. Rapid mixing was used to quickly combine solution A (100 mL) and solution B (900 mL) to carry out the reaction. This ensured that there was adequate mixing and the products formed within 30 s. The heterocyclic nitrogen atom of 4,4′-bipyridine donated a lone pair of electrons during this reaction to Cu^2+^, creating a coordination complex network with the theoretical formula CuC_10_H_8_N_2_C_l2_. The final products were then collected by centrifugation at a speed of 5000× *g* rpm for 15 min and three times in water. The obtained carbon precursors were dried before being pyrolyzed for 2 h at 650 °C in a tube furnace with a heating rate of 1.0 °C min^−1^ while being enclosed in an argon environment. As carbonization progressed, the copper species readily dissociated from the nitrogen-containing backbones, leaving nitrogen heterocyclic rings to fuse and stack around the remaining metal species. 48 h were spent soaking the acquired materials in a 4.0 M HNO_3_ solution to remove any metal impurities. Following repeated deionized water rinse to bring them back to neutral, the dark samples were dried for 24 h at 80 °C in a vacuum oven. Finally, hierarchically porous carbon flowers with highly decorated active oxygen and nitrogen heteroatoms were obtained, which were then kept in a securely closed bottle for subsequent characterizations.

### 3.2. Materials Characterization

X-ray powder diffraction (XRD) analysis was performed using a Rigaku SmartLab diffractometer (Rigaku Corporation, Tokyo, Japan) over the 2θ range from 10 to 70 degrees. The instrument was equipped with a filtered Cu Kα (λ = 1.5406 Å) radiation source and operated at a tube voltage of 40 kV and a current of 40 mA. The Raman spectrum was obtained using a TEO SR-500I-A spectrometer with an excitation source of 532 nm and a laser power of 50 mW. Silicon was employed as a point of reference for calibration. An ESCALAB 250Xi spectrometer (Thermo Scientific, Waltham, MA, USA) was employed to perform X-ray photoelectron spectroscopy (XPS). The process of photoemission was initiated through the utilization of a 300 W monochromatic Al Kα X-ray source with a photon energy of 1486.6 eV. The energy analyzer was configured to maintain a consistent pass energy of 100 eV for survey scans with a 1 eV step size and 20 eV for detailed scans with a 0.05 eV step size of core level lines. The spectral binding energies were adjusted by fixing the carbon C 1s peak at 284.8 eV. XPS peak 4.1 free software was used to fit the core level spectra of processed C 1s, O 1s, and N 1s. Prior to conducting XPS measurements, the specimens underwent a drying process at 110 °C within a vacuum oven for 24 h to eliminate any surface-bound moisture. Sample morphologies were confirmed through the utilization of a JSM-7800 field-emission scanning electron microscope (SEM, 15 kV, JEOL Ltd., Tokyo, Japan) and a JEM-2800 transmission electron microscope (TEM, 200 kV, JEOL Ltd., Tokyo, Japan) equipped with an energy dispersive X-ray (EDX) analyzer. The Quantasorp gas adsorption apparatus was used to collect the isotherm data for nitrogen adsorption and desorption (Quantachrome Instruments, Boynton Beach, FL, USA). The multi-point Brunauer–Emmett–Teller (BET) method was utilized to determine the specific surface areas within a range of 0.05–0.25 P/P_0_. The nonlocal density functional theory (NLDFT) method was applied to calculate pore size distributions based on the N_2_ adsorption branch.

### 3.3. Electrochemical Measurements

The study conducted electrochemical experiments utilizing customer-made Swagelok-type stainless steel setups. Initially, a homogeneous mixture was prepared by mixing active materials (80 wt%), sodium alginate binder (10 wt%), and Super P conductive carbon black (CB, 10 wt%) in deionized water. The resulting slurry was cast evenly onto a copper foil using a doctor blade, which was then subjected to a drying process at 105 °C for 12 h in a vacuum oven. The electrolyte solution was composed of a mixture of ethylene carbonate (EC), dimethyl carbonate (DMC), and ethyl methyl carbonate (EMC) (volume ratio of 1:1:1), with a concentration of 1.0 M NaPF_6_. The counter/reference electrode was composed of pure sodium foil (Sinopharm Chemical Reagent Co., Ltd. Shanghai, China), while a Whatman porous glass fiber membrane was used as the separator. For full-cell measurements, the cathode was commercial Na_3_V_2_(PO_4_)_3_ coated on aluminum foil. The construction of the half-cells and full-cells was conducted within an argon-filled glovebox (MIKROUNA, Shanghai, China) that maintained oxygen and moisture levels below 0.1 ppm. The electrolyte was introduced into the respective half-cells via a micro syringe, maintaining a consistent volume of 100 μL. The loading amount of active materials for all electrodes was 1.0 ± 0.1 mg cm^−2^. The cells in their as-formed state underwent a 12 h aging process while being exposed to the electrolyte solution prior to the gathering of electrochemical data. The study employed multichannel battery testers (CT3002A, LANHE, Wuhan, China) to perform galvanostatic discharge/charge experiments in the voltage ranges of 0.01–3.0 V and 0.7–4.2 V for half-cells and full-cells, respectively, at different current densities spanning from 30 to 5000 mA g^−1^. The experiment involved conducting cyclic voltammetry (CV) at a scan rate of 0.1 mV s^−1^ within the potential range of 0.01–3.0 V. Electrochemical impedance spectroscopy (EIS) was used to measure impedance spectra ranging from 10^5^ to 10^−2^ Hz at the open circuit potential by applying a sine wave of 5.0 mV. An electrochemical workstation (CHI660E, CHI Instruments, Shanghai, China) was adopted to conduct the CV and EIS experiments at room temperature. To guarantee the dependability of electrochemical assessments, replicates were conducted for every specimen. Following 200 repetitive cycles at a current density of 60 mA g^−1^, the cycled half cells were disassembled manually, and the resulting electrodes were rinsed with DMC solvent and dried to eliminate any residual debris. All of the aforementioned post-treatment procedures were carried out within a glovebox under an argon environment. After the cycling process, the cleaned anodes were subjected to analysis via SEM and TEM techniques in order to investigate their post-cycling morphological and compositional characteristics.

## 4. Conclusions

In summary, we have developed a practical synthetic method to prepare novel carbonaceous flowers that possess N/O doping and a multilevel hierarchical porous architecture. Owing to their combined improvements, the N/O-doped porous carbon flowers demonstrate remarkable electrochemical performance as anode materials for SIBs. This includes a high capacity of up to 329 mAh g^−1^, excellent high-rate capability, and an ultralong cycle life of up to 1300 cycles without any discernible decline in capacity, which can be compared with that of other carbon-based anodes reported previously. In addition, a carbon flowers/Na_3_V_2_(PO_4_)_3_ full cell also exhibits high capacity and stable cycling performance. By further modifying the heat treatment procedure and the precursor composition, we will be able to enhance the sodium storage performance of carbonaceous materials in the future. Furthermore, the utilization of the synthetic approach has the potential to create novel opportunities for the sustainable advancement of diverse hybrid materials consisting of carbon flowers and metal oxides, which can be employed in various energy conversion and storage systems, such as fuel cells, batteries, supercapacitors, etc.

## Data Availability

The data presented in this study are available on request from the corresponding author.

## Sample Availability

Samples of the compounds are not available from the authors.
